# Reduced Endothelial Progenitor Cells: A Possible Biomarker for Idiopathic Fetal Growth Restriction in Human Pregnancies

**DOI:** 10.34763/jmotherandchild.20232701.d-23-00014

**Published:** 2023-11-22

**Authors:** Apurva Singh, Shyam Pyari Jaiswar, Apala Priyadarshini, Sujata Deo

**Affiliations:** Department of Obstetrics and Gynaecology, King George's Medical University, Lucknow; Photobiology Division, CSIR-Indian Institute of Toxicology Research, Lucknow

**Keywords:** endothelial progenitor cells, fetal growth restriction, flow cytometry, plasmatic cytokines, novel biomarker

## Abstract

**Background:**

Circulating endothelial progenitor cells (EPCs) may be necessary throughout pregnancy by ensuring proper placentation and embryonic growth. The lack of standardized EPC quantification techniques has prevented conclusive proof of an increase in EPC during pregnancy.

**Objectives:**

The purpose of this study was to determine whether EPC levels change for healthy and idiopathic fetal growth restriction (FGR) pregnancies.

**Materials and methods:**

The study population consisted of 48 healthy pregnant females with no previous history of IUGR (10 in the first trimester, 15 in the second, and 23 in the third), 48 women with pregnancy complicated by idiopathic FGR, and 15 non-pregnant women. By using flow cytometry, EPCs in maternal blood were recognized as CD45dim/CD34/KDR cells. ELISA was used to measure plasmatic cytokines.

**Results:**

We ascertained a progressive rise in EPCs in healthy pregnancies that was apparent in the first but more pronounced in the third trimester. At comparable gestational ages, FGR-complicated pregnancies had impaired EPC growth. Placental growth factor and stromal-derived factor-1 levels in the blood were significantly lower in FGR than in healthy pregnancies, which may have contributed to the degradation of the EPCs.

**Conclusion:**

The count in EPCs might hold considerable promise toward developing a peculiar authentication marker for observing pregnancies, and could be the focus of cutting-edge tactics for the prognosis and treatment of FGR pregnancies.

## Introduction

Fetal growth restriction (FGR) is a pathophysiological repression of fetal growth that results when a fetus fails to reach its full developmental potential. FGR continues to rank among the world's top causes of fetal morbidity and mortality and poses a larger risk to maternal health. A fetus's inability to reach its full growth potential frequently causes pregnancies to be terminated early and increases the risk of both poor neonatal outcomes and long-term health effects. FGR is pervasive everywhere, although it is especially common in underdeveloped nations [1–2]. Global statistics show that approximately 24% of neonates have FGR, which results in 30 million growth-restricted infants each year. However, it is predicted that the rate of FGR in poor nations is around six times higher than in industrialized countries [2]. According to reports, 21% of all live babies in India have FGR [1].

Vascular endothelium, which is generally quiescent in adults, is a crucial attribute in vascular homeostasis. Angiogenesis is a relatively unusual phenomenon in adults apart from placentation, embryogenesis, wound healing, and endometrial repair after menstruation, and the turnover of vascular endothelial cells is generally rather low. Endothelial progenitor cells (EPCs) are circulating cells identified as markers of vascular injury and play a crucial role in adult vasculogenesis and endothelial homeostasis [3–4]. EPCs are secreted from the bone marrow in conditions of stress such as ischemia, myocardial infarction, ischaemic stroke, vascular trauma, sickle cell anemia, vasculitis, and pulmonary hypertension. Amidst these conditions, the level of increased circulating blood returns to its basal level in 48–72 hours. EPCs have proliferative and angiogenic potential and have been implicated in vascular repair through helping restore the integrity of the endothelium [4–5].

It is a well-known fact that various changes take place in the maternal vascular system during pregnancy for proper fetal uptake of nutrients, but the exact role of EPCs is still not entirely understood. The components that govern angiogenesis during pregnancy are not yet known, though it has been documented that it depends on the proliferation and mobilization of EPCs from the bone marrow when sufficient amounts of various growth factors such as vascular endothelial growth factor (VEGF), granulocyte-macrophage colony-stimulating factor (GM-CSF), fibroblast growth factor basic (FGFb), and angiopoietin-1 are released [4,5,6]. By developing and maintaining the vascular network, EPCs play a crucial role in a healthy pregnancy by ensuring successful placentation and fetal growth. Studies conducted to support inconsistent information on the amount of circulating EPCs during pregnancy found that the level of EPCs in the blood of healthy pregnant women was either higher or lower than that of non-pregnant women or remained the same. [7–8]. While Sugawara et al. discovered that there is an increase in EPCs during the second and third trimesters of pregnancy, Gussin et al. concluded that EPCs are present in the peripheral blood during the second trimester of pregnancy [9–10]. Due to the disparities in approach among these studies, the cause of these contradictions must be clarified.

Even though flow cytometry, the most quantitative method to assess EPCs, was used in all cases to estimate EPCs [11], the studies mentioned above disagreed regarding the markers used to define EPCs and the method used to collect the material for estimation of EPCs (complete blood vs. mononuclear cells of blood). The confluence of endothelium markers (kinase insert domain receptor: KDR) and progenitor antigens (CD34, CD133, or both) is required for the flow cytometric quantification of EPCs; nevertheless, standardization is required. It has been proposed that the optimal amalgamation to measure EPCs in the medical environment may be the CD45dim/CD34/KDR phenotype [11] due to the leukocyte marker CD45's increased precision over time. Importantly, very few studies evaluating EPCs during human development included CD45 labeling.

Therefore, we used a CD45 gating method in our investigation to measure EPCs during a typical pregnancy. We also looked at the possibility that women whose pregnancies were hampered by idiopathic fetal growth restriction (FGR), a significant reason for child death and morbidity marked by insufficient placental perfusion, would not experience an increase in blood levels of EPCs [13]. Additionally, the key angiogenic factors known to have an impact on the numbers of EPCs in the blood were assessed at the plasmatic level.

## Materials and Methods

The study was conducted at Queen Mary's Hospital, King George's Medical University, Lucknow, UP, India. The study population consisted of 48 healthy pregnant females with no previous history of FGR (10 in the first trimester, 15 in the second, and 23 in the third), 48 women with pregnancy characterized by idiopathic FGR, and 15 non-pregnant women in the follicular phase of the menstrual cycle. Women who smoked cigarettes or had any illness that influenced the count of EPCs (anemia, preeclampsia, heart diseases, lung diseases, tuberculosis, and carcinoma) were excluded from the study. Women withnormal menstrual cycles who were not on contraceptive pills were included in the non-pregnant controls. Normal pregnancies were those without maternal or fetal pathologies, obstetric complications, and medications that directly effect pregnancy outcome and fetal growth. Normal pregnancies were delivered at 37–42 weeks of gestation [11]. Small for gestational age (SGA), defined as estimated fetal weight (EFW) or abdominal circumference below a certain threshold such as the 10th or 3rd percentile, is most commonly used to suspect FGR [12]. Blood samples (1.5 ml) were collected by venipuncture in EDTA tubes. Women confirmed their participation by signing a consent form, and this study was approved by the institutional ethical clearance committee for human research (ECR/262/Inst/UP/2020).

### Assessment of EPCs in the circulation

**Figure 1.**
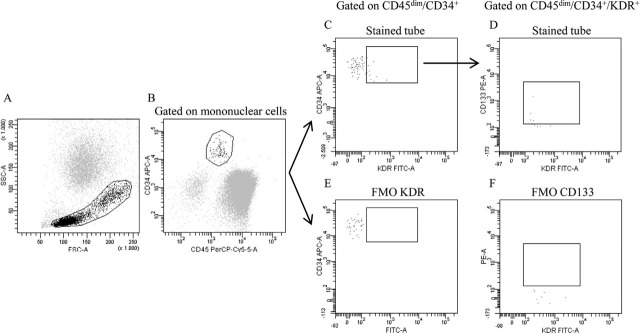
Typical flow cytometry gating technique for circulating Endothelial Progenitor Cells (EPCs) detection. **(A)** To filter out granulocytes, dead cells, and debris, mononuclear cells were gated in a forward-scatter (FSC) side-scatter (SSC) plot by means of blood samples. Then, cells were successively gated depending on the expression of **(B)** CD45dim and CD34, **(C)** Kinase domain receptor (KDR), and **(D)** CD133. FMO (fluorescence minus one) staining was used to create the gates depicting the **(E)** KDR and **(F)** CD133 events. EPCs in circulation were classified as CD45dim/CD34/KDR or CD45dim/CD34/KDR/CD133 cells.

Flow cytometry was used to examine blood samples. Phycoerythrin (PE)-conjugated anti-human CD133, allophycocyanin (APC)-conjugated anti-human CD34, and peridinine-chlorophylle protein complex (PerCP)-Cy5.5-conjugated anti-human CD45 monoclonal antibodies were all incubated for 20 minutes with 200 ml of anticoagulated blood. All antibodies were procured from Sigma-Aldrich. After that, the left-over cells were again incubated with fluorescein isothiocyanate (FITC)-conjugated streptavidin for 20 minutes in the dark to disclose biotin-conjugated anti-human KDR after the erythrocytes were removed [14]. Negative controls were fluorescence-less samples. Samples were examined by means of a flow cytometer and FACSDiva software. Fig. 1 displays a typical flow cytometric investigation. A CD45dim/CD34/KDR or CD45dim/CD34/KDR/CD133 EPC was recognised.

### Measurement of plasmatic cytokines

ELISA kits were used to measure the levels of free vascular endothelial growth factor (VEGF), free placental growth factor (PlGF), and stromal-derived factor-1 (SDF-1). Each step was completed in accordance with the manufacturer's instructions.

### Statistical evaluation

All the analysis was done by using Prism software. For group comparisons, the Mann-Whitney U test was applied. A potential correlation was looked for using the Spearman rank test.

## Results

### EPCs increase in maternal circulation during a healthy pregnancy

**Figure 2.**
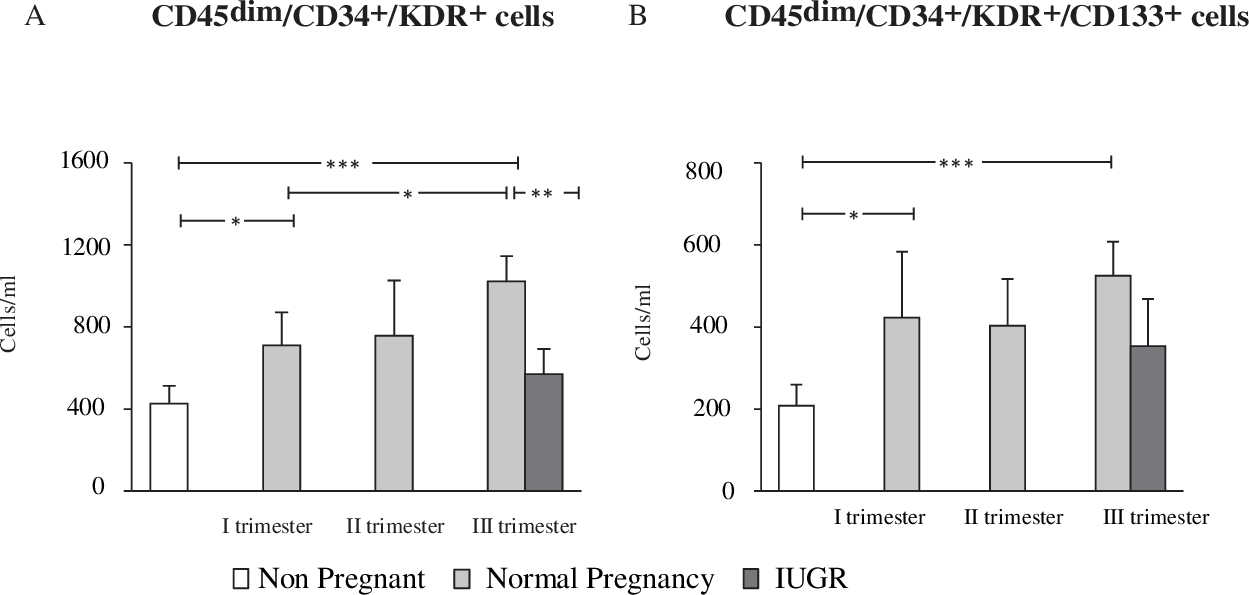
Expression of circulating Endothelial Progenitor Cells (EPCs) during healthy and Intra-uterine Growth Restriction (FGR) complicated pregnancy in maternal blood. **(A)** Pregnancies complicated by FGR at comparable gestational ages resulted in considerably decreased levels of EPCs, which were recognized by flow cytometry as CD45dim/CD34/KDR cells, in the I trimester when differentiated with non-pregnant women and continued to decrease. **(B)** There were no appreciable differences in EPCs classified as CD45dim/CD34/KDR/CD133 cells between FGR and healthy pregnancies. ^*^p < 0.05, ^**^p < 0.02, ^***^p ≤ 0.001.

Circulating EPC concentration, measured as CD45dim/CD34/KDR cells, was 398 ± 79 cells/ml (mean ± SD) in healthy, non-pregnant women. Women in the first trimester of healthy pregnancy had considerably more EPCs as compared to controls (699±158 vs. 398±79cells/ml; *p* = 0.030), as shown in Fig. 2A. With the development of a healthy pregnancy, the EPC count rose; it was more prominent in the third trimester than in the first (1032±792 vs. 699±158 cells/ml; *p* = 0.0398). Similar outcomes were revealed when EPC in circulation was recognized as CD45dim/CD34/KDR/CD133 cells, as illustrated in Fig. 2B.

### Circulating EPCs are decreased in FGR pregnancy

Comparing healthy pregnant women of the same gestational age to pregnant women with FGR, we counted the number of EPCs in the peripheral blood of the FGR patients. There were considerably fewer circulating CD45dim/CD34/KDR EPCs in FGR than in a healthy pregnancy (558 ± 119 vs. 1032 ± 792 cells/ml; *p* = 0.024), with values which were comparable to those seen in non-pregnant women (558 ± 119 vs. 398 ± 79 cells/ml; *p* = non-significant) (see Fig. 2A). Similar conclusions were made when circulating EPCs were characterized as CD45dim/CD34/KDR/CD133 cells; however, in this instance, the difference between FGR and normal pregnancies failed to approach statistical significance (342 ± 24 vs. 517 ± 27 cells/ml; *p* = 0.082), as illustrated in Fig. 2B.

### In FGR pregnancy, plasmatic PlGF levels are significantly decreased

**Figure 3.**
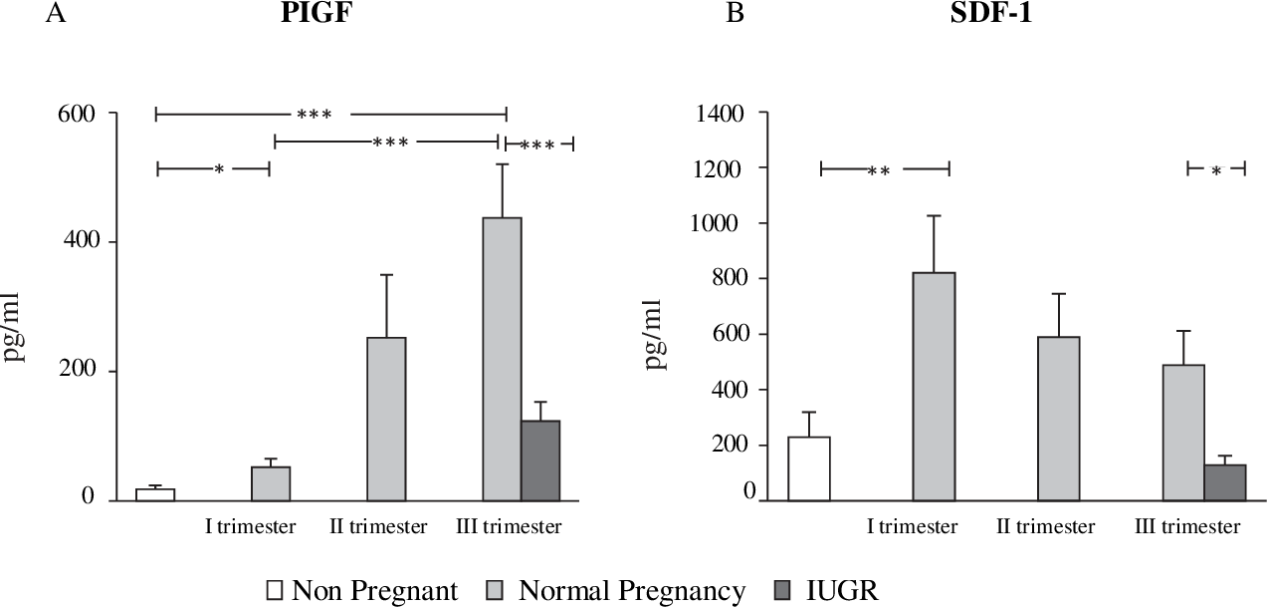
Concentration of Placental Growth Factor (PlGF) and Stromal Derived Factor (SDF-1) in healthy and Fetal Growth Restriction (FGR) pregnancy. **(A)** During healthy pregnancy, PlGF levels gradually increased; however, at the same gestational age, they were considerably lower in FGR than in healthy pregnancy. **(B)** SDF-1 levels grew during normal pregnancy, peaking in the first trimester and gradually decreasing as the pregnancy progressed, although they were lower in FGR than during normal pregnancy. ^*^p < 0.05, ^**^p < 0.02, ^***^p ≤ 0.001.

In order to ascertain whether the variations in EPC figures that transpired throughout healthy and FGR pregnancies might be associated with the levels of soluble factors in the circulation, we assessed the plasmatic levels of angiogenic cytokines, which are known to affect the number of circulating EPCs. As seen in Fig. 3A, levels of PlGF gradually grew throughout a healthy pregnancy and were curtailed in FGR pregnancies, suggesting a trajectory that largely mirrored that of circulating EPCs. The levels of SDF-1 were likewise elevated during a typical pregnancy, as seen in Fig. 3B, with an initial rise in the first trimester that gradually subsided as the pregnancy progressed. FGR pregnancies had lower plasmatic SDF-1 levels than normal pregnancies. The plasmatic levels of free VEGF were less than the 9 pg/ml detection limit in both a regular pregnancy and an FGR pregnancy in all trimesters. None of the plasmatic factors that were evaluated, such as PlGF, SDF-1, or VEGF, were associated with the quantities of CD45dim/CD34/KDR or CD45dim/CD34/KDR/CD133 EPCs.

## Discussion

The main conclusions of our study were that circulating EPCs decrease in an FGR pregnancy while they rise in the maternal circulation during a healthy pregnancy. In an FGR pregnancy, plasmatic PlGF levels are also noticeably reduced.

In our study, we observed that during normal pregnancy, EPCs in the maternal blood gradually increase. This finding was not unexpected, as from the beginning of pregnancy, even in its initial stages, EPC concentrations begin to rise for adequate placental and fetal growth by ensuring the proper formation of the placental vascular network. Our finding was in accordance with the study done by Malluci et al. [15]. Yet an explicit expression of an increase in EPCs has been hindered due to a lack of standardized methods used for the analysis of EPCs. Several studies on the number of EPCs in healthy normal pregnancies, measured either by flow cytometry or cell culture tests done in vitro, disclosed contradictory results [7–8, 16]. In our study, we evaluated EPCs by the flow cytometric method [14], previously published, which recognizes EPCs in blood samples straight away as CD45dim/CD34/KDR cells. It must be noted that such cells might not directly lead to vasculogenesis [4, 17–18], but they are still potential contributors to endothelial homeostasis and angiogenesis [17]. Although much literature on the phenotype of EPCs is still lacking, that which is available supports our result that CD45dim/CD34/KDR serves as an apt antigenic combination to characterize EPCs with reference to precision in detection and clinical usefulness [16]. By employing this technique, we noticed that the level of EPCs in pregnant women's blood starts to rise during the first trimester and increases even more as the pregnancy progresses. We also noticed a decline in the level of EPCs in FGR pregnancies. Similar but not significant findings were reported by Timmermans et al., who used CD133, a stem cell antigen, as a marker to confine EPCs, thus validating that CD45dim/CD34/KDR might be the most suitable phenotype to identify EPCs. This finding agrees with the fact that EPCs do not originate from CD133 cells [19]. The major angiogenic cytokines involved in the mobilization and recruitment of EPC are VEGF, PlGF, and SDF-1 [20–21]. Few authors demonstrated that free plasmatic VEGF was untraceable in both normal and FGR pregnancies, while free SDF-1 and PIGF were present in reduced quantities in FGR as compared to normal pregnancies [22–23]. PIGF is a potent angiogenic factor highly expressed during each stage of gestation in the placenta, playing a crucial role in placental vascularization and fetal growth [20–21]. Hence, the reduced level of maternal circulation found in our study is apt to explain the pathogenesis of FGR. This result was in accordance with other studies as well [22, 24]. SDF-1 is predominantly concerned with the mobilization of EPCs from the bone marrow and stirring the influx of EPCs into the vessel from the circulation when vascular injury and tissue hypoxia occur [19]. It also plays a chief role in placental development. This is the first study that we are aware of to quantify the levels of SDF-1 in maternal circulation, and our novel discovery of noticeably low levels of SDF-1 may be consistent with the aetiology of FGR. This study is the only one to date to assess EPCs in this way in India. Despite the lack of a symbolic link between PlGF or SDF-1 levels and EPC count, our findings may generally imply that reduced accessibility of both cytokines may be a factor in the degeneration of EPC in FGR.

In our study, the illustration of strikingly reduced EPCs is indicative of the fact that EPCs play a role in the development of defective placentas, which characterize FGR. This inference is strengthened by the findings of Sipos et al., who reported that endothelial colony-forming cells derived from FGR cord blood are obscure and dysfunctional, resulting in diminished vasculogenic potential, which could be a cause of placental dysfunction in FGR [25]. These cells correspond to a significant population of EPCs possessing the ability to proliferate and angiogenic activity in vivo [26]. As per this conclusion, it has also been described that cultured mobile angiogenic cells derived from the blood of pregnancies complicated with FGR upon delivery are diminished and show impairment in their capacity of migration [27], and those derived from flow cytometry are reduced in the cord blood of FGR pregnancies [28]. Our study has major advantages in terms of the requirement of a very small amount of blood samples, easy availability of blood, and speediness in execution when compared with other methods. These characteristics may make the examination of EPCs in circulation a suitable technique that could be successively replicated during the monitoring of pregnancy, signifying that the circulating EPC levels could certainly be deployed as potent biomarkers in FGR pregnancies.

As a result of the exponential decrease in EPC levels during FGR pregnancies and functional damage posed by their reduction, EPCs might serve as the objective of novel therapeutic strategies designed to avert FGR-related unfavorable pregnancy results. Statin therapy may be useful for women with FGR pregnancies since it is known to increase circulating EPCs, enhance mobilization, migration, and survival, and stimulate PIGF [17, 29]. The use of statins during pregnancy is contraindicated due to their potential teratogenicity, which may be brought about by the disruption of cholesterol synthetization. Cholesterol and its derivatives are considered essential components for fetal development, and they are involved in the synthesis of steroids and cell membranes. A study by Chang et al. proposed that statins may be safe when used during pregnancy because there was no association with congenital anomalies [29]. In this case, we would propose that FGR may be considered a pregnancy complication that could respond favorably to statin medication once the safety of statins in pregnancy is confirmed.

## Conclusion

We showed that during healthy pregnancies, EPCs grow in the maternal circulation, and that this rise is hindered in pregnancies complicated by FGR. This study is the only one to date to assess EPCs by flow cytometry in India. EPCs in the blood may be counted using flow cytometry to create a helpful biomarker for diagnosing and tracking pregnancies complicated by FGR, as well as in the perspective of future EPC-targeted therapeutic strategies for these serious pregnancy complications.

### Highlights

- CD45dim/CD34þ/KDRþ serve as apt antigenic combination to characterize EPCs
- Low levels of SDF-1 may be consistent with the aetiology of FGR
- EPCs might serve as the target of novel therapeutic strategies
- FGR may be considered a pregnancy complication that could respond favorably to statin medication once the safety of statins in pregnancy is confirmed.
